# Positive body image: a qualitative study on the successful experiences of adolescents, teachers and parents

**DOI:** 10.1080/17482631.2023.2170007

**Published:** 2023-01-29

**Authors:** G Tort-Nasarre, M Pollina-Pocallet, Y Ferrer Suquet, M Ortega Bravo, M Vilafranca Cartagena, E Artigues-Barberà

**Affiliations:** a Calaf Primary Care Center, SAP Anoia-Gerència Territorial Catalunya Central, Institut Català de la Salut (ICS), Calaf, Barcelona, Spain; b Department of Nursing and Physiotherapy, Faculty of Nursing and Physiotherapy, University of Lleida, Lleida, Spain; c AFIN, Research Group and Outreach Centre, Universitat Autònoma de Barcelona, Barcelona, Spain; d Bellpuig Primary Care Center, Diputació, Lleida, Gerència Territorial Lleida, Catalan Health Institute (ICS), Barcelona, Spain; e Research Support Unit Lleida, Fundació Institut Universitari per a la recerca a l’Atenció Primària de Salut Jordi Gol i Gurina (IDIAPJGol), Lleida, Spain; f Cappont Primary Care Center, Lleida, Gerència Territorial Lleida, Catalan Health Institute (ICS), Barcelona, Spain; g Department of Medicine, Faculty of Medicine, University of Lleida, Lleida, Spain; h Department of Nursing, Faculty of Health Science and Welfare, University of Vic-Central University of Catalonia (UVIC-UCC), Manresa, Spain; i Balafia Primary Care Center, Lleida, Gerència Territorial Lleida, Catalan Health Institute (ICS), Barcelona, Spain

**Keywords:** Positive body image, qualitative study, adolescents, parents, educational personnel

## Abstract

Purpose Body image encompasses body-related self-perceptions and personal attitudes. Dissatisfaction with body image during the early stages of adolescence is negatively related to self-esteem and other health problems. A few publications focused on positive body image and directly related to the experiences and interactions of adolescents themselves. To explore positive body image in adolescents and describe the familial and educational factors that contribute to its development.Methods A qualitative study was conducted. Purposive sampling was used, and 9 adolescents, 6 families, and 8 teachers participated in semi-structured interviews, which were then theme analyzed. Results Self-care, body acceptance, confronting messages that attack body image, and the influence of social media have been identified as emerging themes in adolescents’ positive body image experiences. Therefore, the pubertal period, family values, fostering, and educational actions as well as media literacy were identified as factors promoting self-esteem and positive body image in the family and educational environment. Conclusions Their parents also expressed aspects such as those that contribute to the development of healthy self-esteem, confidence, and positive body image. Alternatively, the teachers indicated educational activities to work on self-image and self-esteem when faced with situations of concern in the classroom.

## Introduction

**Adolescence** is a transitional period between childhood and adulthood, and it is divided into early adolescence (from 10 to 14 years old) and late adolescence (from 15 to 19 years old) (Unicef Nations Cildren’s Fund, 2011). This period is characterized by significant physical, psychological and social changes that affect the adolescent’s growth and can pose challenges for developing and/or maintaining positive body image (Parent et al., 2003; Rogol et al., 2002). In addition, these biological, cognitive and psychosocial changes tend to be more prominent and abrupt during adolescence in comparison to adulthood (Markey, 2010) as adolescents are positioned to accept or reject their maturing body and participate in self-care or dangerous behaviours (Sundgot-Borgen et al., 2019). Various social agents have strong interactions with adolescents, such as their family, peers, groups, educational institutions and the media, which helps to construct a system of values and beliefs that are the basis for the adolescent’s self-confidence (Rosenberg, 1989). The quality of these relationships has a key role in their psychosocial development (Mota & Matos, 2014).

Body image (BI) is conceptualized as the internal representation that someone has of their external appearance (Grogan, 2016) and encompasses self-perceptions related to the body and personal attitudes, including thoughts, beliefs, feelings and behaviours (Cash & Smolak, 2011; Sands, 2000). At the same time, body image is a complex phenomenon that includes several components with gender, ethnic and sociocultural influences (Cash, 2012; Cash & Smolak, 2011).

Alternatively, body dissatisfaction is defined as a person’s negative thoughts and feelings about their body. It is related to negative assessments of the size, shape and weight of the body, and generally involves a perceived discrepancy between the person’s assessment, their body and their ideal body (Grogan, 2016; Tiggemann et al., 2011). Likewise, dissatisfaction with body image during the early stages of adolescence has been related to lower self-esteem and predicts depressive symptoms, a higher body mass index, less physical activity, clinical eating disorders, a poorer quality diet, disordered eating (i.e., diet behaviours based on trends, food and use of ingredients for emotional regulation), behaviours aimed at losing weight (through diets, food restrictions and modification of dietary habits), which at the same time foster problems with the distortion of self-image (Vaquero-Cristóbal et al., 2013). Also, some authors have associated certain internal factors with body dissatisfaction and the concept of the body (Heidelberger & Smith, 2018), the perception of beauty and ideals of appearance transmitted by society and the media (Meier & Gray, 2014), the attitude towards the self and towards others (Heidelberger & Smith, 2018) and gender differences and perspectives (Ramos Valverde et al., 2010). Other authors have identified the media together with family and friends as part of the external factors that influence BI (Heidelberger & Smith, 2018; Ramos Valverde et al., 2010; Vaquero-Cristóbal et al., 2013). Tort-Nasarre et al. (Tort-Nasarre et al., 2021) suggest that the construct of body image in adolescents has multiple meanings and that it does not necessarily involve dissatisfaction when there is a mismatch between the real and desired image. The concept of body image is established in a much more complex way than simply through the messages received by the mass media and the pressure experienced from friends. The family and the school environment also play a determining role in terms of protective factors when work is done to provide tools to help them develop self-esteem, assuredness and confidence.

There are plenty of studies on body dissatisfaction in adolescents all over the world and also in Catalonia (Pollina Pocallet et al., 2019; Pollina-Pocallet et al., 2021), however during recent decades, in the context of research into body image there has been a call to focus on positive body image and its links to wellbeing (Tylka et al., 2018). Positive body image has been conceptualized as a multidimensional construct that represents love and respect, acceptance and appreciation of individuals and feeling comfortable with their body, independently of their actual physical appearance, as well as their ability to interpret messages in a way that protects the body (i.e., internalizing positive messages, rejecting or reformulating negative messages (Tylka & Wood-Barcalow, 2015b). The response to this perspective has been to develop a multidimensional conceptualization of positive body image among adults (e.g., Tylka & Wood-Barcalow) (Tylka & Wood-Barcalow, 2015a) but among adolescents it has still been scarcely studied (Frisén & Holmqvist, 2010) even though, as mentioned previously, the formation of positive attitudes and perceptions of the body is important during adolescence both for girls (Wertheim et al., 2011) and for boys (Ricciardelli & Cash, 2012).

## Aim

Due to the relative scarcity of research on positive body image and the need to clarify the interactions between some protective and risk factors related to this construct during adolescence, this qualitative study aims to explore positive body image in adolescents and describe the familial and educational factors that contribute to its development. The specific questions that guided the investigation are: What characterizes positive body image in adolescents? Which factors in the familial and educational environment influence positive body image in adolescents?

## Methods

### Design

We used a qualitative descriptive design, an approach that is suited to reaching a deeper understanding of practice in applied disciplines (Colorafi & Evans, 2016). This design is especially pertinent when the goal is to understand the participants’ perspectives and experiences (Morse & Field, 1995).

### Participants

Purposive sampling was used with the aim of recruiting good informants who meet the research objectives (Moser & Korstjens, 2018). The participants were part of three groups: students, teachers and parents of students at a school in Bellpuig, Lleida, Catalonia, Spain. The inclusion criteria for participants were being students in the Compulsory secondary education, being considered by their peers as someone with high self-esteem, good body image and assuredness. Being teachers at the school and considered by the students as references. Being parents of students chosen by their peers as references.

The school nurse helped to recruit the participants. The recruitment process was complex as there were three groups of participants to recruit: adolescents, parents and teachers. To recruit the adolescents, a questionnaire (ad hoc) was given in which the students were asked to write the names of three classmates that they thought had high self-esteem, assuredness and good self-image, and another questionnaire (ad hoc) to write down the names of the three most important teachers of their school year. Both questionnaires were anonymous. 324 adolescents responded to the questionnaires, based on the list obtained a definitive list was drawn up and the adolescents who received the most votes from each year group were asked to participate, as well as the teachers who were the most highly rated. Lastly, the parents of the students who received the most votes were invited to take part in the study. Finally, 9 students, 8 teachers and 6 mothers/fathers were chosen.

One of the team’s researchers (MPP) invited the candidates to participate in the study via telephone. Those who accepted were sent the informed consent and information sheet for the study that was collected by the same researcher.

### Data collection

The data were collected using semi-structured interviews with the students, teachers and parents. The team of researchers created a set of interview questions based on the existing literature and adapted to the three profiles of participants. During the interviews, the participants were encouraged to add additional information on their experience. The interviews were carried out by three members of the team: MPP, GTN and EAB from July 2021 to March 2022. The interviews were carried out firstly via video call through Teams due to the lockdown situation as a result of COVID-19 and the latest ones were carried out face-to-face with the teachers and parents. During the interviews, notes were taken on the non-verbal communication of the participants. The interviews lasted approximately 30–45 minutes. All the interviews were recorded on a tablet and were transcribed. All the interviews were anonymized, assigning them an alphanumeric code. They were encrypted and stored in a dissociated manner on a secure server at the Institution. The transcribed interviews were also returned to the participants for their approval. All the participants accepted the transcription.

### Data analysis

The thematic analysis method was followed (Braun & Clarke, 2006) and it was organized using Atlas-ti version 7. GTN and EAB carried out the data analysis. They began to familiarize with the data to obtain a complete view of them. Later, meaning units of the text were identified. Similar expressions were put together, sub-themes and later main themes were created. In doing so, they remained faithful to the participants’ expressed perspectives (Patton, 1990). Quotes were also extracted to illustrate the results, indicating the participant’s code at the end of each of these. Data saturation was reached when we detected that no relevant new information was emerging (Saunders et al., 2018).

### Rigour

The study complied with credibility, transferability, dependability and confirmability which ensures the trustworthiness of the qualitative research (Johnson et al., 2020). Credibility was ensured due to the constant review process of the process of analysis. Transferability was ensured through the detailed description of the phenomenon in sufficient detail to be transferable to other settings and people. Dependability was ensured in this study due to the review by each of the researchers of the process of analysis as well as the final product. Confirmability was assured through the reflective work of each researcher who made field notes during the interviews to offer the most objectivity possible in the data collection and analysis. We completed the Consolidated Criteria for Reporting Qualitative Research (COREQ) (Tong et al., 2007). This study was approved by the ethics and clinical research committee at the Institut d’Investigació IDIAP Jordi Gol under the code 20/154-P.

## Results

23 participants took part in the study: 9 students between 12 and 16 years old (77.8% female and 22.2% male), 8 secondary school teachers who were references for the students (62.5% female and 37.5% male) and 6 parents of the students (83.3% female and 16.7% male) (Table I).

**Table I.** Characteristics of the participants.

		Adolescents						
			Sex		Academic year		Number of siblings	
A1			Female		1st year ESO		Four siblings, she is the youngest	
A2			Female		2nd year ESO		Two siblings, she is the youngest.	
A3			Male		3rd year ESO		Five siblings, he is the youngest	
A4			Female		4th year ESO		Three siblings, she is the middle child	
A5			Female		2nd year ESO		Two siblings, she is the oldest	
A6			Female		4th year ESO		Two siblings, she is the oldest	
A7			Male		4th year ESO		Two siblings, he is the oldest	
A8			Female		4th year ESO		Two siblings, she is the youngest	
A9			Female		1st year Baccalaureate		Two siblings, she is the youngest	
		Parents						
			Family relationship		Age		Marital status	
P10			Mother		50		Separated	
P11			Mother		47		Separated and with partner	
P12			Mother		47		Separated	
P13			Mother		52		Separated and with partner	
P14			Father		51		Married	
P15			Mother		56		Married	
P16			Mother		44		Married	
	Teachers							
	Age	sex		Studies		Subject		school year
T17	59	Female		Masters		Entire curriculum		6th year of primary education
T18	57	Female		Masters		Mathematics		1st year of ESO
T19	43	Male		Spanish		Spanish language and literature		4th year ESO
T20	42	Male		Business		Social sciences and IT		3rd and 4th year ESO
T21	43	Female		Chemistry		Technology, physics and chemistry		1st and 4th year ESO
T22	45	Female		Translation and interpreting		English		1st year ESO
T23	55	Male		Environmental sciences		Biology and geology		3rd year ESO
T24	52	Female		English and German language		English		2nd year ESO

The participants are coded as follows: A1-A9: Adolescents, P10-P16: Parents and T17-T22: teachers.

In the data from the interviews 2 themes and 8 sub-themes were identified. See Table II

**Table II.** Themes and sub-themes.

Theme	Sub-theme
Experiences regarding the adolescents’ positive body image	Doing self-care Acceptance of one’s own body Confronting messages that attack body image Influence of social networks
Promoting factors in the familial and educational environment	Pubertal timing Family values and actions that promote self-esteem and positive body image Educational actions that generate self-esteem and positive body image Media literacy

### Theme 1: experiences regarding the adolescents’ positive body image

The adolescents express the aspects that characterize positive body image and they are summarized in the following sub-themes: doing self-care, acceptance of one’s own body, confronting messages that attack body image and the influence of social networks.

#### Doing self-care

The majority of the adolescents talk about actions aimed at caring for the body, such as doing sport and having a good diet:

I am well looked-after. I play tennis 5 days a week. I like it a lot. I do lots and lots of sport. A3.

They value the advantages and benefits that looking after the body brings to their physical and psychological wellbeing:

I have always considered sport to be something you need, not for getting thin, but for distracting yourself and getting rid of everything that’s happened when you have a very dark and busy day, doing sport airs you out and goes really well. A6.

In addition, they identify diets that are not appropriate for their development:

I think that when there are people who are fine and they go on diets to be thinner because they are obsessed with their body, I think that it’s not good, if you eat well and do sport it’s better. A1.

And they give healthy advice to friends who want to get thinner and discourage risky behaviours:

Sometimes, when one day they aren’t feeling good, they want to lose weight… then most of us say that there’s no need, that they’re fine and not to do silly things like stopping eating, vomiting… and all of that. And that if they have to do it, that they do it right by eating and doing sports. A5.

#### Acceptance of one’s own body

The adolescents show aspects related to the acceptance and appreciation of their own body. Many of them like their body:

I like my body, I wouldn’t change anything right now, I am how I would like to be. A1.

Others explain that you have to accept yourself as you are physically and not put too much importance on the body:

You have to accept yourself as you are and you mustn’t put much importance on the body and try to be like another person to be better, rather that, sometimes, what you think is better. A4.

There are also adolescents that believe that it is more important how you are internally as a person:

I put a normal amount of importance on it, not a lot and not very little. The most important thing is how you are, your personality. A1.

In this manner, the adolescents indicate that you have to be secure in yourself:

If you don’t feel well with yourself others won’t like you either and you have to appear secure in yourself. A3.

good self-esteem as a protective measure:

I believe that you can be physically not so perfect, but if you have good self-esteem and you like yourself, you’ll see yourself as perfect and it won’t matter to you what other people say about you, alternatively, maybe you’re fine but you have very low self-esteem, and you’ll give the slightest hurtful comment they make more importance than the 30 positive ones they make. A5.

They also highlight the relationship between accepting the body and accepting yourself as inseparable aspects:

I believe that if you feel good with your body, you’ll also feel good with yourself because it is part of you. And I think that it is important but not very, we shouldn’t give it much importance because there are people who are obsessed with their body, and it’s not necessary. A4.

Others explain the need to normalize body image and accept different canons of beauty and health:

Normalizing that being fat or being very thin is not a very bad thing. Maybe, let’s see, being very, very fat might be harmful for the health, but equally we have to normalize everything, that it’s not bad that someone is fatter or that someone else is thinner. A1.

However, a few adolescents express aspects related to the appreciation or acceptance of the image of others:

I think that you have to respect everyone. A3.

Alternatively, there are young people who don’t fit into the socially established canons of beauty. They feel the need to hide their body and adopt avoidant behaviours when they are exposed to the rest of their peers. These behaviours are determined by the opinion that they themselves have of BI and they differ between boys and girls:

It is very important, because girls sometimes more than boys tend to obsess more over the physique and follow certain ideals. The boys maybe don’t talk about it, but inside they feel it too. A4.

The teachers also observe differentiating details between the boys and girls regarding a body that is not within the pre-established social canons:

The boys that are a little beyond what is normal might be chubbier, you do see them as being very shy, very much so, maybe extremely shy more than a fat girl, alternatively, with the girls if they’re a little outside of the common pattern I see them as somewhat disinhibited. T22.

But on the other hand, being different to the rest of the group is challenging and a concern for many of them. Faced with this experience, the teachers describe the individual adaptive responses of these students when they feel exposed to their peers, for example when they have to speak to an audience:

You see that they are a little embarrassed going to speak to an audience because they don’t feel confident in their body. T24.

or how they hide the body to not show it to their peers:

They wear looser things, or sweatshirts when it’s hot and they don’t even want to take off their coat (…) because either they have boobs because they’re fat or they have a belly. T24.

The teachers mention how the process of adaptation by the group is for those who have a different physical image to the rest:

There are groups that wouldn’t accept them because they are dressed differently, because they are different and there are groups that would accept them because they’re bringing other things as well as the way they dress. T20.

##### Confronting messages that attack body image

Some adolescents consider receiving messages from peers as a positive reinforcement:

My friends always tell me that they think I have a very good body. It isn’t important but I like it. When you receive these positive comments from your friends it makes you feel better and makes you like yourself more. A3.

But the influence of friends is not always positive, they also explain about messages between friends that attack the body image of others, causing upset between them:

They go along with them because they are very thin or because they don’t have boobs, for example… there are kids that tell them this. A1.

The teachers also highlight situations that are threatening for body image between peers:

Some girls are having a bad time due to comments that the boys are making. That they’re fat, that they don’t have breasts, that they have a fat bum. That’s what the boys say. The boys say things to the girls and there are even girls that were considering not going to the swimming pool or not wearing a bikini because of what the boys might think about their body. T20.

In the face of these threatening messages, the parents and teachers assess high self-esteem and confidence in adolescents as key factors for not letting themselves be influenced:

If you have a good base and good self-esteem it doesn’t have to affect you so much either. But if you’re someone who has very fragile moments maybe what they say affects you more. P11.

At the same time, the adolescents identify different strategies for dealing with the negative messages they receive from their peers such as selective listening:

I’m a person who doesn’t listen to harmful criticism very much, because it isn’t useful. You have to listen to the good ones to have good self-esteem and self-image. A6.

defending the weaker peer:

There were some boys who were calling another person names. They were calling them fat and such. They were starting on their body weight. Us girls always told them to be quiet, it was all the girls against these boys. A4.

or even helping them with strategies that they have learned by experiencing similar situations:

I went to help this person, because when they did it to me, I didn’t know how to defend myself either so I think that what’s needed when they’re doing that to you is to stick out your chest and face them so they see you, so they can’t bring you down. A5.

##### The influence of social media

The influence of social media with regards to body image is another of the elements that they talk about. It is worth underlining that the adolescents are fully aware of the effect of the messages they receive about beauty ideals on social media:

There are lots of comments talking about the physique on social media and this very much affects us teenagers, who are the people who use it most. A2.

and their consequences when they don’t comply with the beauty canons broadcast by the mass media:

I think we’ve reached a point where everyone gives it too much importance, because everyone also focuses on it a lot and brings down other people due to the appearance that society has given us of how a person has to be or how they have to stop being, people on TV, influencers. They give us an appearance that everyone wants to achieve and if you don’t have a certain build you can’t manage to have it. People have become so used to the fact that if someone is different, the only thing they do is bring them down because they are different. A5.

#### Theme 2: promoting factors in the familial and educational environment

In terms of the manner in which parents and educators talk about issues related to the appearance, the following sub-themes were identified: Pubertal timing, Values and actions that promote self-esteem and positive body image, Educational actions that generate self-esteem and positive body image and Media literacy.

##### Pubertal timing

In many cases, the parents and educators know what it means to go through these vital stages characterized by a process of physical, psychological and emotional change. They indicate that they know about aspects of the adolescent stage:

It changes a lot, there are animal changes on physiological levels, they’re animalistic the changes that happen. T23.

For the teachers and parents these physical changes have an emotional impact and cause a lack of emotional regulation:

it’s a stage with lots of changes, they aren’t balanced, as soon as they’re happy they’re angry, they can feel happy with themselves or not. It’s a stage of lots of imbalances and little acceptance and of rage and getting angry, being angry. Little acceptance of rules, or of themselves, it’s like they’re a little angry with the world… T22.

And it also affects self-esteem and self-knowledge:

They’re really in a process of change, so what happens? They don’t feel well with their mind or with their body because they’re in a process of change. Of course, they don’t yet really know where to go and then their self-esteem is affected. P10.

And they identify consequences related to social interaction,

It’s a topic that matters so much to them and they really look after their image, excessively so I think rather than too much, but of course it’s their whole world right now, the social relationships, fitting into the world, and they value it excessively. I find that there are lots of problems related to food that are unnecessary. T21.

They also have lots of problems, I think, psychological problems, problems like anorexia. It’s that now, social media and everything like that is making it very bad. P12.

They also indicate the influence of friends and the fact of following a uniform pattern of dressing and thus feeling integrated into the group:

Friendships do a lot! It’s that for them at this stage of life, friendships are more important than parents and being another one in the group is essential for them. T24.

I see that their external style is always a mono-topic. Nobody goes outside the norm, I don’t know any 1st year ESO student that has their own style, in other words, they all wear the same type of T-shirt. What I see is that there is one style and nobody departs from that. T18.

##### Family values and actions that promote self-esteem and positive body image

Another topic that was identified in the data was the transmission of values in the family environment. These values are defined as aspects that facilitate the children having positive body image and high self-esteem. The parents highlighted the affective link, help and accompaniment:

There is an esteem link, the family link, the link that has to transmit values, values for making your bed, values for cooking, values for beliefs I mean… that’s what a family is, not just transmitting certain values. I think that it has to transmit all of them, the more the better. P10.

They teach them to find solutions based on parental communication:

What I always repeat to them is that if they ever see a problem, they find themselves trapped, they find that they don’t know what to do, as much as it seems very complicated, very difficult and very hard to them, that they should talk to me about it because just the fact of explaining half of the problem works and then, maybe between the two of us we can find the solution that one person alone can’t find. P13.

Accompaniment that is guided, and they explain how they put limits on their children and the reason for doing so,

I put limits on them. Or rather you reach a point where it’s “Right, that’s it”. I’ve tried to reason everything… or I say something, I don’t agree, I don’t see it like that, whatever… but if I really think that it is better for them and the other thing isn’t good, if necessary, in the end I’m capable of saying “that’s it, because I said so” because I’m reasoning everything to you and I’m giving a basis for it and you’re not paying attention to me, you’re not listening, so that’s it. P13.

Teaching them to have their own style, their own opinion and not letting themselves be influenced by others:

At home certain values have been given, you’re clean, you’re polite, you dress in the style you like, but you dress well and from there everyone has their own style. P13.

This accompaniment and values are received and interpreted by the children as they explain:

They’ve never told me to stop eating, that you’re fat or to eat less. They’ve never started on me and my body. A4.

Physical appearance at my house is not very important, it’s more important to be a good person. A6.

The adolescents explain how their parents teach them skills and points of view in the face of negative comments that attack the physical appearance:

My Mum and Dad told me and everyone has told me you have to learn to defend yourself, you can’t lower your head and let them bring you down because none of us are better, nobody is better than anyone. Then at home they also helped me, they told me that I had to defend myself without lacking respect. A5.

They are families that generate healthy relationships with self-esteem and self-image. The parents express, among the strategies that they talk about, they believe that it is crucial to be present in the day-to-day and maintain good communication links:

I am always very much in contact with the children. We do lots of things together, we talk a lot, I go in their room, they come in mine, I mean that at my house there are no closed doors. I think that they tell me about lots of things, I’m sure they don’t tell me everything, or anywhere close, but they tell me lots. P10.

In addition, the families also talk about activities and actions that they do to educate and work on the self-esteem and positive self-image of their children. Such as working on self-acceptance and confidence in yourself:

We’ve prioritized feeling confident in yourself, or loving yourself as you are, accepting yourself and giving others the image of who you really are inside. I have always given them certain very clear parameters around what to do in lots of situations. For self-confidence and self-esteem, I think that all that makes self-esteem, feeling confident. P13.

Giving positive reinforcements:

Giving them positive reinforcements with the things that you see that they can highlight and those that they do not, so bringing out the good. Make them see what they’re worth, so they go looking for their path towards things that can be good. P11.

Looking after yourself:

You have to have a good concept of yourself, you have to look after yourself. Looking after yourself doesn’t mean you have to be thin, that you have to be all muscular. P13.

Knowing how to decide and being responsible for the consequences:

You have to make a decision, you have to evaluate it and once you’ve evaluated it, you have to accept that maybe it won’t please everyone and that you’ll have to be responsible for your actions. P13.

##### Educational actions that generate self-esteem and positive body image

The teachers explain in detail their educational aim and how this is determined by professional teaching values: respect, consideration, visibility and not judging. The importance of these aspects for the development of self-esteem:

Respect, consideration, giving each student the place that corresponds to them, meaning that they aren’t invisible in the classroom, even if they aren’t always visible. They’re all part of that group and this is very important for self-esteem. T17.

Evaluating them:

My job is also to evaluate them very positively. T18.

Establishing a good channel of communication:

I talk to the students a lot when I can. Whenever I can. T24.

It is important to highlight that the teachers identify certain characteristics in the adolescents with low self-esteem:

A person who is insecure, introverted, people who are negative and for whom it is difficult to get themselves up, when so many young people sometimes tell you “It’s hard for me to get up in the morning but I’ll make the effort. T19.

As a trait characteristic of the adolescent age in current society:

They have low self-esteem, because it’s protected them a lot and this has made it so that any form of effort grinds them down, makes them suffer, they get exhausted. From this culture of effort, trying, falling and getting back up again and all that I don’t think so: they don’t have too much of it. T20.

In addition, they highlight how self-esteem is a key element for functioning throughout life and carrying on despite having little integration of a culture of effort:

A person with self-esteem will carry on going. T17.

They describe teaching strategies when faced with adolescent students with low self-esteem,

With students with low self-esteem what I do, what I try to do is give them an important place in the class, but as important as that of any other student. T17.

giving elements because they believe in themselves and encouraging them to tackle challenges:

Reinforcing self-esteem, with the feeling of always giving them encouragement and forward movement and you’ll come out of it and not doing the opposite: “you’ll do nothing with your life”. T21.

giving confidence through communication:

Speaking with them: to give them confidence because they talk to me about what is happening, and then when they explain what is happening, trying to look for solutions within what I can do. T20.

These teaching actions have the reward of feeling like they help the adolescent:

There are lots of teachers who help with lots of students who don’t find themselves, who don’t see themselves or have a negative image of themselves, a physical as well as mental image, we go along with them and, at times, as well, if the thing isn’t very serious sometimes they come out of this type of dark hole. T23.

The teachers at the school carry out activities aimed at educating the adolescents on self-esteem and emotional regulation:

We did an activity that was called Great and it was about saying positive things, not about buttering them up or telling lies, but saying positive things about yourself, the teachers, your peers… well activities like that. T21.

These teachers find themselves in situations in the classroom in which they have to work on positive body image and thus avoid dissatisfaction or conflict and carry out activities aimed at this such as:

The other day I did an activity where I put images of the kinds of girls that the boys follow and that they like and such and afterwards I put the opposite, those images of the boys that the girls follow. Then I had them stand up in front of the class and I asked: boys are you the same as these boys that come up here on social media? No, so don’t ask the girls to be the same as these girls. It was an exercise in seeing that one thing is day-to-day reality, and the other is how they want to appear. T20.

By comparing images between one and the other they see it is a visual thing and this stays with them there. T20.

Sitting in a circle and expressing how they feel or how they feel from what the others make them feel with them, of how they treat them, how they stop treating them and comparing themselves, from now on… speaking about it among them all in small groups to talk about it and how to be able to solve this and how you make the other, with your own reactions, can make the other feel better. T20.

#### Media literacy

Another emerging theme that the parents and teachers mention is the use that the adolescents make of social media and how they influence BI. For the educators and parents, media literacy and education is a challenge. For example, they realize the effects that it has for young people listening depending on which type of music that entails implicit patriarchal values:

It’s this patriarchal culture that we have in our society that, as much as we try to fight and fight, there is always this background of social media, music… everything that affects adolescents. This music leaves the girls on the ground. And the girls listen to this music and sing it and then you say it’s just lyrics, no no, but this gets into you and you end up feeling the same as the boys that follow it. And this very negatively affects them and they don’t realize it as much as we try to educate, educate, educate… we have all this background. T20.

Or of how they mirror the success of the youtubers:

If I have a youtuber as a model, and a youtuber making 4 videos earns a living and a very good one, why do I need to make an effort? If I’m an instagrammer what I’m doing is uploading photos of how I dress, what I visit, the places I go, etc., with this I’m earning a good living why do I need to make an effort, why do I have to do a degree, a training course, it’s not worth it, so this low-effort society, I think it also affects them. T19.

Even so, both the teachers and the parents look to teach the critical analysis of the information and what they receive on social media:

I’m sick of telling them not to look for information where we don’t know that the source is reliable or they might be tricked, or going to look for videos that don’t explain anything good to you, don’t look at this, do a search. Guiding them a bit. T23.

*They’re becoming aware of the manipulation of the images they receive on social media*:

They see it on a tablet, remind themselves with a TikTok: here there are lots of filters what an embarrassment, this girl with such a filter, look on Google and compare. I think that it’s going to make the kids open their eyes, that all that is a lie, it’s cinema, it’s a film: they’re wearing make up, it’s all… it’s all a lie. T22.

## Discussion

This study has explored positive body image in adolescents in Catalonia (Spain) as well as the perspective of parents and educators. In particular it contributes data from the adolescents considered by their peers as having high self-esteem, confidence and good body image, the teachers who are references for these same students and their parents, an aspect that contributes to the in-depth and detailed results on positive body image at this age. In this regard, the formation of positive body attitudes and perceptions is an important development task during adolescence. In particular, the influence of the education received from parents and friends has been studied (Frisén & Holmqvist, 2010), but there is a lack of studies on the influence of the school environment, an aspect that can be essential and which is covered in the current research.

### Experiences regarding positive body image

With regards to the aspects that characterize positive body image, many adolescents describe doing self-care as a beneficial aspect that brings them physical and psychological wellbeing (Wood-Barcalow et al., 2010). In addition, it is worth pointing out that they are capable of detecting unhealthy habits such as unhealthy diets or excessive physical activity and giving healthy lifestyle advice to friends when they detect that they are doing these practices. This reinforces a protective role towards body dissatisfaction associated with the development of eating disorders or psychological stress (Añez et al., 2018; Dhillon & Dhawan, 2011).

This study also highlights the association between high self-esteem, confidence and self-image. In this regard, some participants prefer being good people even though their body is not perfect, and having confidence in yourself and being capable of not paying attention to negative comments on your appearance. These are aspects that are reinforced by the work of Holmqvist et al. (Holmqvist & Frisén, 2012) as protective attitudes.

In addition, another aspect to highlight is the acceptance and appreciation of their own body as inseparable aspects and ones that make up the identity (Holmqvist & Frisén, 2019). However, the study shows that there are also adolescents that hide their body and adopt avoidant behaviours when they perceive that they do not have the body that they would like, seen from the perspective of the educators. The study has not identified the consequences of the appreciation of the body of others. However, it is possible to confirm this through the follow-up of the qualitative study on these adolescents.

The influence of friends is not always positive and at times they find themselves in situations where their self-image or that of their peers feels threatened. In these cases it must be emphasized that the adolescents with good confidence defend the weak ones, or rather they acquire an attitude of selective listening and do not let themselves be influenced by these comments. These abilities to face negative comments may suggest aspects for development in programmes that promote health and the prevention of body dissatisfaction.

The adolescents are fully aware of the influence of social media and of how it can condition their self-image and self-esteem (Burnette et al., 2017) and body dissatisfaction (Calado et al., 2011; Mooney et al., 2009). The results indicate that those adolescents with good self-esteem and confidence in themselves are also more critical when faced with the messages received from the mass media. Therefore, helping the adolescents to understand this influence in the school and family environment can be highlighted as a recommendation for the development of healthy body image and self-esteem.

### Promoting factors in the familial and educational environment

The parents and educators selected by the students themselves have a direct influence on the way in which the adolescents perceive their self-image. One of the important things that they do to help them to perceive a healthy self-image and self-esteem, among others, is talking about these topics with them, therefore building support bridges. In this manner, the accompaniment of the parents and educators, when they know the characteristics of the adolescent stage and are capable of understanding it, are preventive measures that must be considered in programmes that educate about health.

In relation to family values, we have shown how the vast majority of parents have a good communication link with their children, a link of esteem and a safe environment. In addition, they impose limits when it is necessary and put in place guidelines to be followed. At the same time, the children evaluate the messages received from their parents as help to make them grow up. These families have healthy interpersonal relationships, many parents take actions aimed at working on positive self-image and self-esteem, they can affirm with these results that they may be crucial elements for the development of healthy body image. Alternatively, they are educationally complex aspects that may be included in interventions such as “parent school” (Sharpe et al., 2013).

Another aspect that is worth highlighting from the study and which has been scarcely studied is the role of educators (Ciao et al., 2018; Sundgot-Borgen et al., 2018). The data from the study provide new information specifically with regards to the context of the promotion of healthy body image. For example, these schoolteachers take actions aimed at working on self-esteem and confidence, resolving situations of conflict between peers. The educational aim is based on educating the adolescents. In addition, the influence of social media can increasingly lead to body dissatisfaction (Burnette et al., 2017), the study underlines that the teachers need to place emphasis on media education to help the adolescents to have a critical attitude towards the negative messages from social media.

To finish, the adolescents’ successful experiences related to body image cannot be understood without considering the influence of their families and teachers. These results suggest inclusive interventions, not only from the school environment, but also in the community sphere that may have a positive impact (better acceptance, fostering of self-esteem, expansion of perspectives on beauty ideals, normalization of concerns between parents and educators) despite the direct influence exercised by friends and social media during adolescence.

## Limitations

This study has various limitations. The first is that it can only be transferable to similar social and cultural contexts and adolescents or young people of the same age band. The sample is small and, therefore, it is not representative of all adolescents with a similar profile. The context in which the study was carried out was limited to schools in a specific area in Catalonia (Spain) with specific sociodemographic characteristics. A wider sample of participants from different educational, family and social backgrounds may reinforce this study. It is also important to highlight that in a qualitative descriptive study it is impossible to quantify the experiences of the participants. Finally, our study only includes the perspective of adolescents, families and teachers, and it is possible that a qualitative discussion methodology such as a focus group may provide new information on the topic. Likewise, a more complete image may arise if the perspective of school nurses and healthcare workers were included.

## Conclusions

The study provides a perspective on positive body image in adolescents, families and teachers in an area in Catalonia (Spain). The adolescents communicated their experiences and perspectives related to positive body image such as the actions aimed at the care of the body, the acceptance of the body and the normalization of differences and security with oneself. Their parents also expressed aspects such as those that contribute to the development of healthy self-esteem, confidence and positive body image in this crucial stage of development and maturation, placing emphasis on family activities and values. Alternatively, the teachers indicated educational activities to work on self-image and self-esteem when faced with situations of concern in the classroom, the fostering of a critical attitude and media literacy on the messages received on social media.

This research provides a complete description of aspects relevant to positive body image addressed in the current social and cultural context in which the adolescents are immersed.

## Implications for clinical practice

The diversity of experiences and points of view given in this study reflects the complexity of the construct of body image that still has aspects that can be explored further. The results cast light on certain multifactorial components of body image in adolescence to be taken into account when designing and implementing educational programmes for the promotion of healthy self-esteem, confidence and body image in adolescence in order to prevent eating disorders. The role of nurse as well as teacher in the community context plays an important role in the promotion of health on this topic. Inclusive programmes that are carried out by various health professionals may help these adolescents to prevent eating disorders. In addition, it is also important to highlight that the active participation of adolescents in programmes on body image in the school setting is necessary. Also, it is necessary to carry out interventions directly in families or with teachers in order to make them aware of the importance of creating a favourable environment for positive body image.
